# Proof of Concept Application of Hydrophilic Interaction Chromatography for Direct Online Disruption of Lipid Nanoparticles, Intact mRNA Analysis, and Measure of Encapsulation Efficiency

**DOI:** 10.1021/acs.analchem.5c00565

**Published:** 2025-04-01

**Authors:** Jonathan Maurer, Matthew A. Lauber, Szabolcs Fekete, Mateusz Imiołek, Camille Malburet, Marc François-Heude, Davy Guillarme

**Affiliations:** † School of Pharmaceutical Sciences, University of Geneva, CMU-Rue Michel Servet 1, 1211 Geneva, Switzerland; ‡ Institute of Pharmaceutical Sciences of Western Switzerland, University of Geneva, CMU-Rue Michel Servet 1, 1211 Geneva, Switzerland; § mRNA Center of Excellence, Analytical Sciences, Sanofi, 1541 Avenue Marcel Mérieux, 69280 Marcy l’Etoile, France; ∥ Waters Corporation, 34 Maple Street, Milford, Massachusetts 01757-3696, United States; ⊥ Waters Corporation, CMU-Rue Michel Servet 1, 1211 Geneva, Switzerland

## Abstract

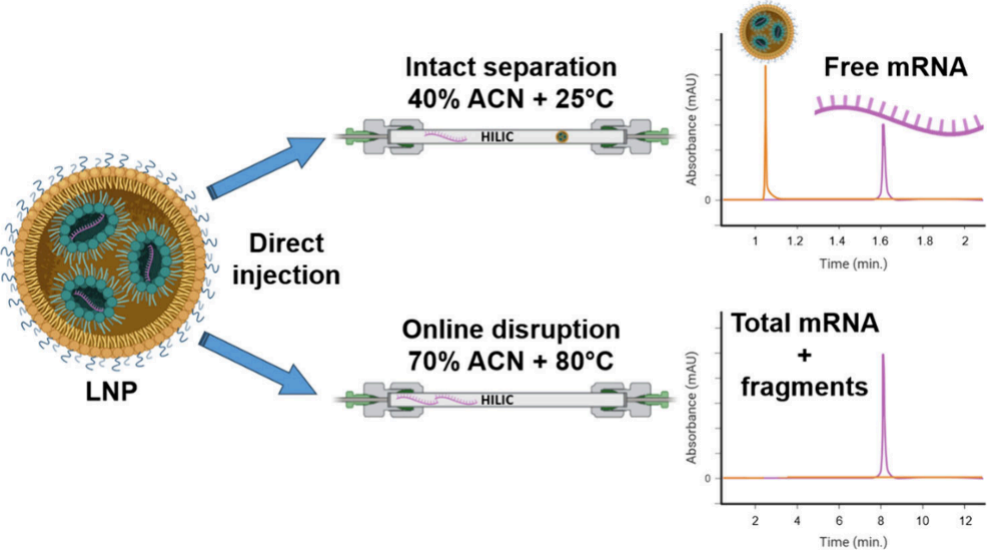

Lipid nanoparticles (LNPs) are a key platform for delivering mRNA vaccines and therapeutics with numerous innovative drugs under development. However, characterizing these complex and unstable products remains challenging. Developing fast, reliable methods to assess critical quality attributes (CQAs) of the mRNA component is crucial for ensuring the safety and efficacy of these medicines. Currently, evaluating key CQAs, such as mRNA integrity and encapsulation efficiency, often involves a labor-intensive manual extraction protocol, which requires LNP disruption prior to analysis. However, these additional offline steps contribute to mRNA degradation and measurement uncertainties, highlighting the urgent need for rapid and effective methods capable of performing an online LNP disruption. Hydrophilic interaction chromatography (HILIC) might offer a promising solution to address this need. Due to the presence of high concentrations of organic solvent and the possibility to work at elevated temperatures, HILIC might enable on-column disruption of LNPs while preserving the full integrity of the mRNA payload, facilitating a streamlined characterization process. To evaluate this, we developed two proof of concept HILIC methods. The first one disrupts LNPs and retains the mRNA payload using a high percentage of organic solvent and elevated temperatures. The second one, relying on milder conditions, retains only the unencapsulated mRNA, which can be used to evaluate the encapsulation efficiency. Both methods were used on Comirnaty and Spikevax vaccines and on Sanofi’s in-development mRNA product as model samples. Our preliminary findings suggest that HILIC holds potential for online LNP disruption, mRNA integrity assessment, and encapsulation efficiency analysis. They also highlight the limitations of small-pore-sized columns currently available on the market.

## Introduction

Messenger RNA represents a novel class of therapeutics that can instruct cells to translate encoded proteins for the treatment and prevention of disease. mRNA drugs work by delivering synthetic mRNA sequences into cells, where they are translated by ribosomes to produce proteins. This approach offers several advantages, including rapid development and production, flexibility in targeting various diseases, and the ability to elicit robust immune responses.^1^

Ensuring the identity, integrity, and content of mRNA drugs is critical for their effectiveness and safety.^2^ Identity testing confirmed that the correct mRNA sequence is present, ensuring that the translated protein was effective for the intended purpose. Purity testing ensures that the mRNA drug is free from contaminants such as double strand RNA, residual DNA, in vitro transcription reaction components, and other impurities that can arise during the manufacturing process, to avoid triggering an unwanted immune responses or inducing toxicity.^3^ Accurate measurement of the mRNA concentration is also crucial to ensure that the correct dosage is administered. Under-dosing may indeed result in insufficient protein production, leading to ineffective treatment, while overdosing can cause excessive protein production and could increase the risk of adverse effects.

One specific form of mRNA purity testing consists of evaluating RNA integrity. This testing verifies the proportion of mRNA molecules that are intact, which is important because the presence of degraded nucleic acids could decrease protein production. Assessing mRNA integrity is also key to ensuring batch-to-batch consistency in manufacturing and adhesion to regulatory standards. Integrity is most commonly evaluated by capillary gel electrophoresis or liquid chromatography.^4−6^

One of the most common ways of formulating mRNAs is by encapsulation with lipids to form a lipid nanoparticle (LNP).^7,8^ Such mRNA-LNP drug products are now used to deliver COVID-19 vaccines.^9^ Thus, before the RNA payload of a LNP can be tested for integrity or content, the LNP must generally be deformulated.^10^ As of now, the use of separation-based approaches (e.g., capillary gel electrophoresis, liquid chromatography) to evaluate a sample’s integrity requires laborious sample preparation procedures.^11,12^ As an example, Raffaele et al. showed the use of microchip capillary electrophoresis for the integrity of loaded mRNA in LNPs using offline disruption by surfactants.^5^ Only one recent study from Imiołek et al. describes direct LNPs online disruption for payload analysis, using size exclusion chromatography (SEC) with surfactants in mobile phases.^13^ While this method holds promise, the use of surfactants directly in SEC columns might reduce their lifetime and prevent their hyphenation with a mass spectrometer. As a result, new approaches that avoid surfactants and manual disruption are highly sought after.^14^

Hydrophilic interaction chromatography (HILIC) has been extensively used for small oligonucleotides analysis.^15−17^ With its standardized use of a high organic solvent concentration and elevated temperatures, it might be an effective chromatographic method for online disruption of LNPs and subsequent characterization of large mRNA payloads. On the other hand, milder HILIC conditions, based on higher percentages of aqueous mobile phase and lower temperatures, might present options for a fast analysis of mostly intact LNPs and allow an orthogonal evaluation of encapsulation efficiency.

To qualitatively assess the potential of HILIC for mRNA analysis, a proof-of-concept chromatographic method was developed to retain mRNA cargo following the disruption of the encapsulated LNP drug products. The method was developed by using commercially available Comirnaty and Spikevax vaccines. We first found optimal HILIC conditions for the measurement of large mRNAs integrity. Then, we added harshly disruptive conditions to induce online disruption of the LNP samples. Subsequently, a water gradient was applied to separate the RNA payload components according to their length and potential poly A tail or sequence variants. Additionally, we explored a second method with milder conditions to assess free mRNA. These methods were evaluated using a complex mRNA drug product provided by Sanofi with a known integrity and encapsulation rate. On this sample, the two HILIC approaches yielded promising results, aligning with those obtained from orthogonal methods. While further comprehensive validation is required to confirm these findings, this technical note highlights HILIC as a potential alternative for online LNP disruption, subsequent mRNA integrity analysis, and encapsulation efficiency assessment of mRNA-LNP drug products in just two analytical runs without any sample pretreatment.

## Experimental Section

### Chemicals and Samples

Ultrapure water was obtained from a Milli-Q purification system from Millipore (Bedford, MA, USA). RNase-free water and Triton X-100 Surfactant were purchased from Sigma-Aldrich (Buchs, Switzerland). LC-MS grade acetonitrile and Tris-EDTA RNase-free buffer (20×) were purchased from Thermo Fischer Scientific (Reinach, Switzerland).

The method was developed and tested on the Comirnaty vaccine (08/2022, NDC: 59267–0304–1) purchased from Pfizer Inc. (USA), BioNTech SE (Germany), on the Spikevax vaccine (09/2022, NDC: 80777–279–05) purchased from Moderna Inc. (USA), and on an in-development mRNA drug product provided by Sanofi (France).

### Hydrophilic Interaction Chromatography

HILIC analysis was performed using a GTxResolve Premier BEH amide column (1.7 μm, 300 Å, 2.1 mm × 50 mm) from Waters (Milford, MA, USA) and a Waters ACQUITY UPLC I-class system, equipped with a 10 μL flow through needle injector, a binary solvent manager equipped with a 100 μL mixing chamber, and a photodiode array detector. UV signals were monitored at wavelengths of 230 and 260 nm. Data acquisition and instrument control were performed by Empower 3 software (Waters). Injection volume was set to 1 μL, and samples were not diluted prior to injection to avoid any LNP disruption. Autoaddition injection mode was employed, with an injection of 4 μL of ACN before and 5 μL after the sample. The HILIC mobile phase A (MPA) consisted of 20 to 100 mM ammonium acetate (without pH adjustment) filtered through a 0.22 μm filter prior to use. Mobile phase B (MPB) consisted of 100% acetonitrile (ACN). For the disrupting conditions, the gradient was run at 0.3 mL/min and at a temperature of 80 °C, starting with 70% MPB from 0 to 1 min, decreasing to 40% MPB from 1 to 2 min, and further decreasing to 30% MPB from 2 to 12 min. For the intact separation conditions, the gradient was run at 0.6 mL/min at a temperature of 25 °C and at a gradient from 40 to 25% MPB in 2 min.

## Results and Discussion

### Intact LNP Disruption and mRNA Integrity Testing

LNPs are highly sensitive to factors such as temperature, pH, and solvents. Therefore, LNP disruption often involves the use of an organic solvent or salt to disrupt the compacted lipid structure. This is followed by centrifugation to recover the precipitated mRNA. Alternatively, Triton X-100 or Brij 58 surfactants can be used to deform the LNPs. However, these additional steps are time-consuming, and the extra sample handling can increase the risk of mRNA degradation.

HILIC gradients that start with a high percentage of organic solvent and an elevated temperature could be employed to disrupt LNPs. Lipids, due to their hydrophobic nature, interact weakly with the HILIC stationary phase (unlike in reverse-phase liquid chromatography) and are eluted in the dead time. In contrast, the highly hydrophilic mRNA payload is effectively retained under the HILIC conditions. By gradually increasing the water content in the mobile phase, the mRNA payload can be eluted and its components separated based on their polarity. In this study, we aimed to evaluate whether the online stress applied during HILIC is sufficient to fully disrupt the LNPs.

**Figure 1:**
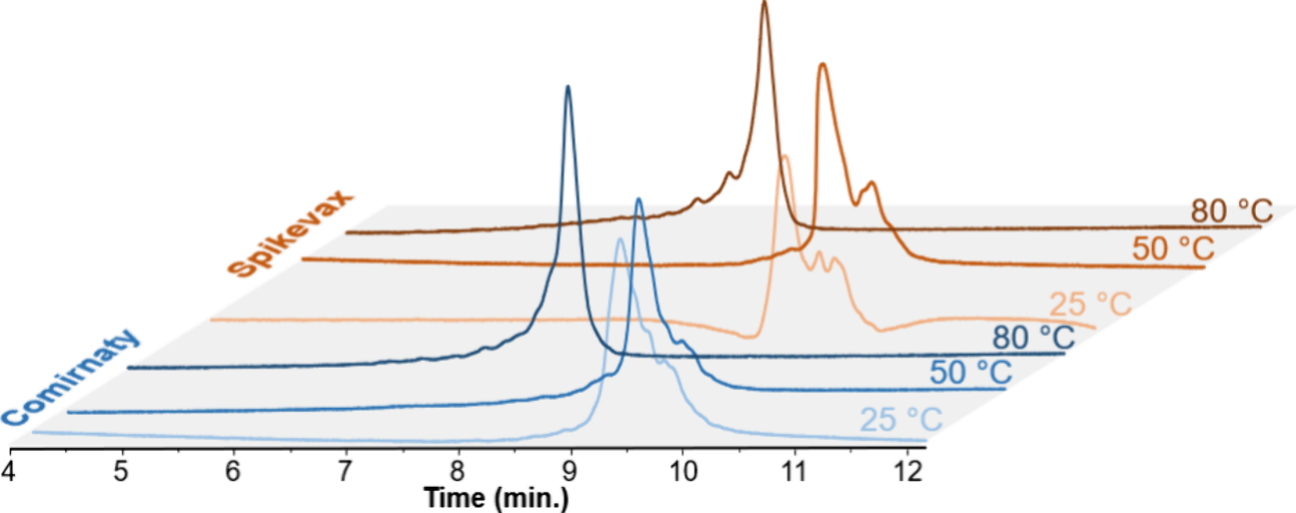
Chromatograms obtained from the analysis of Comirnaty and Spikevax vaccines at various temperatures. Analyses were performed using a starting ACN concentration of 70%, followed by a gradient of 1% ACN/min for 10 min. Mobile phase A was composed of 100 mM ammonium acetate.

To test this hypothesis, we employed relatively harsh HILIC conditions and evaluated the method using Comirnaty and Spikevax vaccines. At the start of the gradient, the ACN proportion was set to 70%, as an optimal balance between maintaining the solubility of mRNA and promoting LNP disruption. This composition was held constant for 1 min before being quickly lowered to 40% ACN. Then, a gradient of 1% ACN/min was applied over a 10 min period. MPA was composed of 100 mM ammonium acetate. Three different column temperatures (25, 50, and 80 °C) were evaluated to check the method effectiveness. As shown in Figure 1, the method showed significant online degradation of the LNP structure, with encapsulated mRNA being released and eluting between 8 and 11 min. The various species that composed the mRNA payload were effectively separated on the HILIC stationary phase. However, due to low resolution, detailed integrity analysis was difficult. It was noted that the online disruption of both vaccines was primarily driven by the presence of organic solvent since an appreciable mRNA signal was observed at all tested temperatures. However, the mRNA profiles differed markedly. At 25 and 50 °C, numerous late-eluting peaks were observed, likely due to these temperatures being below the melting points of the two mRNA vaccines, allowing various conformers to be detected. In contrast, analysis at 80 °C yielded an unfolded mRNA form (temperature beyond the melting point of any self-folded structures),^18^ representing an integrity measurement under conditions of minimal residence time and neutral pH. Interestingly, the linearized mRNA did not exhibit an increased retention time, despite the anticipated effect of an expanded polar surface due to unfolding.

**Figure 2:**
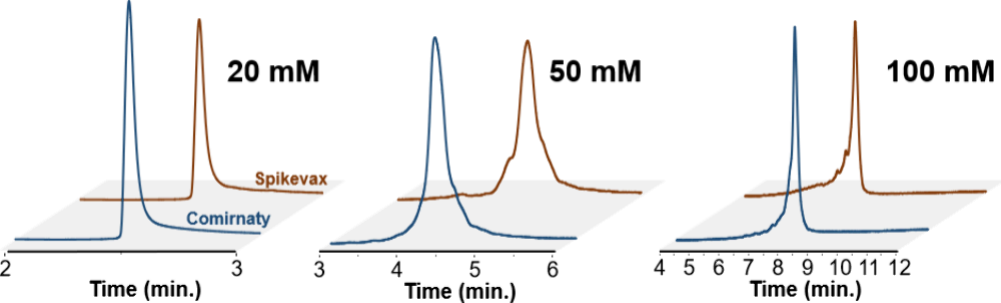
Impact of the ammonium acetate concentration on retention and resolution for Comirnaty and Spikevax vaccine mRNA payloads. Analyses were performed with disrupting conditions, starting ACN concentration of 70% followed by a gradient of 1% ACN/min for 10 min at 80 °C.

We also tested different concentrations of ammonium acetate to evaluate the impact on the retention and integrity profile of the Comirnaty and Spikevax vaccines under disrupting, high temperature conditions. The tested conditions are summarized in Figure 2.

Our results indicate that increasing the salt concentration enhances the retention and improves resolution. Consequently, early eluting peaks are better resolved using 100 mM ammonium acetate, which was selected as the reference method. Ammonium bicarbonate and ammonium formate were also tested but did not improve the resolution (data not shown). Methanol was not considered as the organic modifier, as it consistently afforded lower peak capacity and retention compared to ACN (data not shown). This behavior was expected as methanol is a protic solvent that will compete with water to hydrate the stationary phase, thus limiting the formation of water layer at the surface of the stationary phase^19^ and the strength of direct hydrogen bond analyte adsorption. It is noteworthy that none of the tested mobile phases were able to provide efficient separation of large mRNA. Even with extremely shallow gradients approaching isocratic conditions, we failed to significantly enhance separation between the early eluting peaks and the main mRNA, due to severe peak broadening. To limit the impact caused by the water content in the sample, we worked to limit the injection volume to only 1 μL and employed an autoaddition injection mode (POISe injection), with an injection of 4 μL ACN before and 5 μL after the sample.^20−22^ Unfortunately, it failed to significantly enhance separation between the early eluting peaks and the main mRNA. As a compromise between resolution and peak broadening, we settled on a gradient slope of 1% ACN/min. This limited resolution may be due to the relatively small average pore size (300 Å) of the HILIC column, which restricts the diffusion of large mRNA molecules, induces pore exclusion effects, and limits the kinetic performance. As a result, achieving separation of closely related mRNA species having more than 1000 nucleotides remains challenging with the currently available HILIC columns. The development of columns with larger pore sizes is anticipated to address this limitation and validate the method. To assess whether HILIC might compete with other established methods for integrity measurement, such as ion pairing reversed-phase liquid chromatography (IP-RPLC), microchip capillary electrophoresis (mCE), or mass photometry, new HILIC stationary phases with pore sizes that match the hydrodynamic radii of large mRNA species are needed.

### Evaluation of LNP Disruption

**Figure 3:**
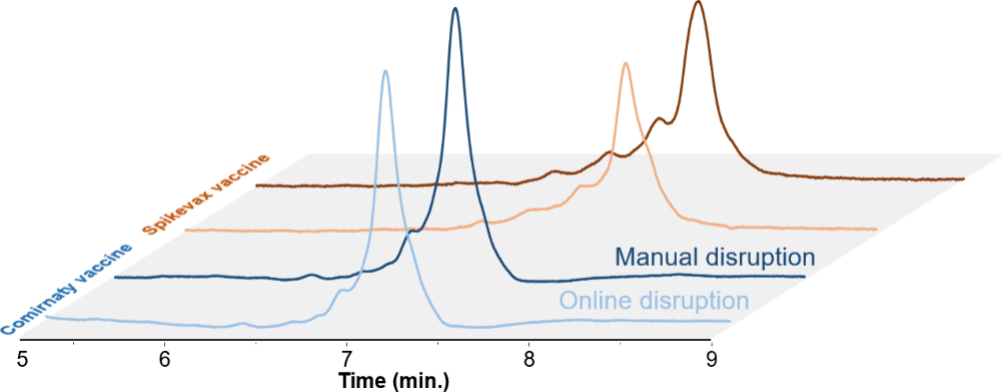
Chromatograms obtained for the analysis of the Comirnaty and Spikevax vaccines using manual or online disruption. Manual disruption consists of a sample diluted 10 times in T4TE20X buffer, while online disruption consists of a sample diluted 10 times in TE20x. Analyses started with 70% ACN for 1 min, followed by a gradient from 40% to 30% ACN in 10 min at 80 °C.

To evaluate the efficiency of online disruption, the results were compared with those obtained with a manual disruption of the vaccines. The manual process involves diluting the samples 10-fold in a commercial tris-EDTA 20× (TE20x) buffer, to which 4% (v/v) of Triton X-100 surfactant was added (T4TE20X). Tris improves mRNA stability at elevated temperatures and pH 7,^18^ EDTA improves mRNA recovery, and Triton X-100 surfactant disrupts the LNP structure. The manually disrupted samples were then qualitatively compared to samples diluted 10-fold in TE20x alone, and both were analyzed using the denaturing method, as shown in Figure 3. Due to the high dilution of the samples, the injected volume was increased from 1 to 4 μL to ensure adequate sensitivity.

The results revealed no visual differences between manual and online disruption methods. Disruption efficiency was evaluated by comparing the peak areas of the online and manually disrupted samples. The online disruption method was 89.3% and 87.1% of the manual disruption’s efficiency for Comirnaty and Spikevax vaccines, respectively. This indicates that the LNPs were nearly completely disrupted, with most of the mRNA payload successfully released under the disrupting HILIC conditions. It is possible that column residence time, organic solvent proportion, and temperature could be further optimized to increase the disruption efficiency to values closer to 100%. The profiles of early eluting peaks were also qualitatively comparable between the two methods. For the Comirnaty vaccine, early eluting peaks accounted for 13.4% and 16% in the online and manual samples, respectively, while for Spikevax vaccine, they represented 31.6% and 33.5%, respectively. Integration limits are presented in Figure S1. It is worth noting that these samples of drug products were analyzed after their expiry. As a result, the integrities measured in this study likely do not represent those of patient quality vaccines.

RNA purity was evaluated using the ratio of absorbance at two UV wavelengths (260 and 230 nm). A ratio between 1.9 and 2.1 indicates pure RNA, while a ratio lower than 1.9 suggests incomplete disruption due to LNP scattering at shorter wavelengths.^23^ For the Comirnaty vaccine, the ratio was 2.16 for online disruption and 2.13 for manual disruption. For the Spikevax vaccine, the ratio was 2.04 for online disruption and 2.07 for manual disruption. These results further confirm the disruption of the LNPs under harsh HILIC conditions.

While the profiles for manual and online disruption were visually comparable, showing similar early eluting peaks, individual peaks were somewhat poorly resolved. This lack of resolution hinders accurate identification and quantitation of the different mRNA species observed, which could be addressed through the design of new HILIC stationary phases. Proper validation of the method using columns with pore sizes that match hydrodynamic radii of large mRNA is needed to confirm the potential of HILIC as an effective orthogonal alternative to other techniques, such as IP-RPLC and mCE.

### HILIC for Encapsulation Efficiency

mRNA encapsulation efficiency (EE) is an assessment of the amount of mRNA successfully encapsulated within an LNP. It is calculated as the ratio of free mRNA to total mRNA. Free mRNA is measured directly from the analysis of intact drug products under conditions that preserve the LNP structure, while total mRNA is determined from disrupted drug products. To accurately measure free mRNA amounts, the HILIC conditions must be mild to avoid disrupting the LNP structure. The encapsulation efficiency is then calculated by dividing the peak area of free mRNA obtained under intact HILIC conditions by the peak area of total mRNA measured under disrupting HILIC conditions, using eq 1.
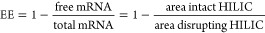
1The intact HILIC conditions were developed to reduce sample exposure to organic solvent. The starting organic solvent percentage was lowered to 40% ACN (compared to 70%), the temperature was decreased to 25 °C (instead of 80 °C), and the flow rate was increased from 0.3 to 0.6 mL/min to minimize residence time. The gradient time was also shortened to 2 min. While this fast method was insufficient for separating mRNA species (and thus unsuitable for integrity testing), this limitation was acceptable, since the primary objective was to measure free mRNA. Using these optimized intact conditions, we analyzed the Comirnaty and Spikevax vaccines in their ready-to-administer form.

**Figure 4:**
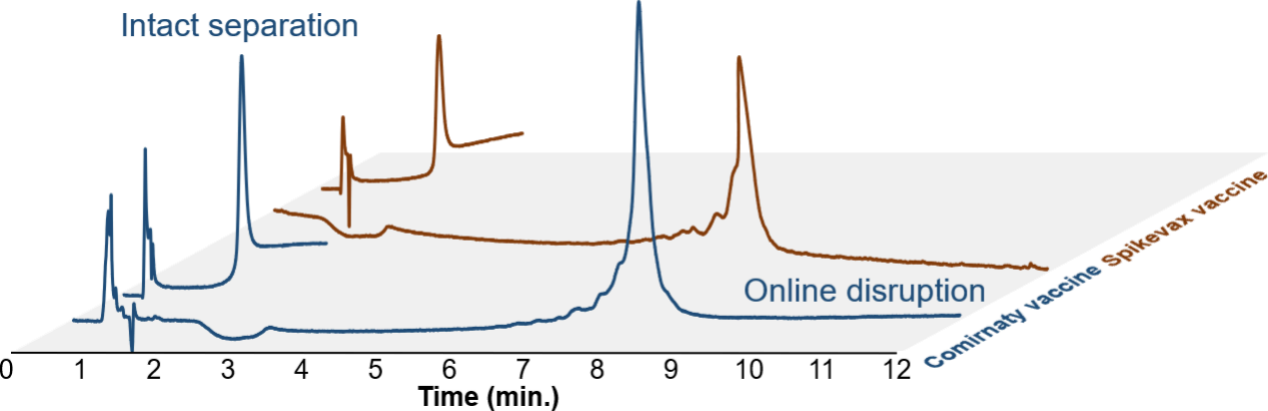
Chromatograms obtained for the analysis of Comirnaty and Spikevax vaccines using intact and disrupting HILIC conditions to obtain free and total mRNA levels, respectively. Intact HILIC conditions started with a linear gradient from 40% to 25% ACN in 2 min at a flow of 0.6 mL/min and a temperature of 25 °C. Disrupting HILIC conditions started with an isocratic composition of 70% ACN for 1 min, followed by a linear gradient from 40% to 30% ACN in 10 min at 80 °C.

Results presented in Figure 4 show the free and total mRNA levels for the two vaccines. Under intact HILIC conditions, LNP degradation was significantly reduced, and the proportion of free mRNA was <20% for both vaccines. The calculated encapsulation efficiencies (EE) were 82% and 85% for Comirnaty and Spikevax vaccines, respectively. These values were slightly lower than the ones reported in literature, which are between 88 and 92%.^24,25^ Again, it is worth noting that the samples were analyzed after their expiry. Consequently, the encapsulation efficiencies reported here do not represent those of commercially available products. Another possible explanation is that even under intact HILIC conditions disruption of more weakly formulated LNPs might occur. Given their inherent instability and high sensitivity to environmental factors, these nanoparticles might still be partially compromised, leading to an overestimation of free mRNA levels. As such, this technique could potentially be used to screen formulations and for quick process development testing. Through the use of an ultrashort 20 mm long column to reduce pressure and shear forces and further reduction in residence time, it could also be possible to refine this new approach. Confirmation of the results obtained on multiple well characterized mRNA-LNP samples is needed in the future to propose HILIC as a potential alternative to other methods for the evaluation of encapsulation efficiency, such as RiboGreen.

## Analysis of a Complex, In-Development mRNA Drug Product

**Figure 5:**
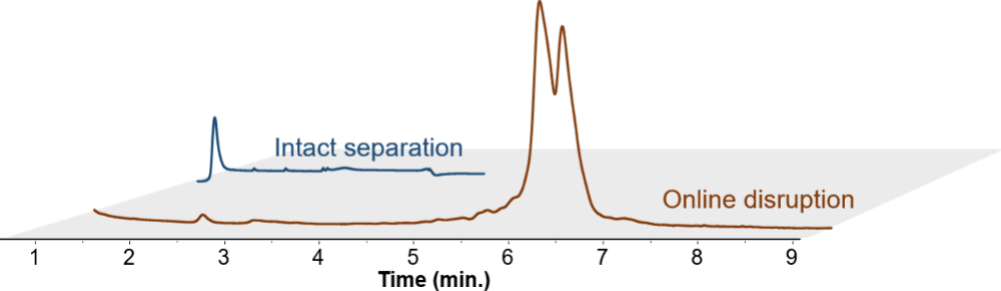
Chromatograms obtained for the analysis of an in-development mRNA vaccine (Sanofi) using intact and disrupting HILIC conditions to obtain free and total mRNA levels, respectively. Intact HILIC conditions started with a linear gradient of 40% to 25% ACN in 2 min at a flow of 0.6 mL/min and a temperature of 25 °C. Disrupting HILIC conditions started with an isocratic composition of 70% ACN for 1 min, followed by a linear gradient from 40% to 30% ACN in 10 min at 80 °C.

To assess the applicability of the method, we evaluated the integrity and encapsulation efficiency of an early stage mRNA drug product from Sanofi with a known composition and encapsulation efficiency. The sample is composed of two mRNA species of 1845 and 1860 nt. Results for mRNA integrity, obtained using the disrupting conditions, and encapsulation efficiency, measured under intact conditions, are presented in Figure 5.

Under online disruption conditions, the two >1800 nt mRNA species were separated, despite their small size difference of only 15 nucleotides. This separation, driven by hydrophilicity, supports the potential of HILIC as an orthogonal method to IP-RPLC for large mRNA analysis. Encapsulation efficiency calculated for this sample was 95.7%. For the same sample batch, EE measured using RiboGreen and ion-exchange chromatography were 94% and 96.7%, respectively. By providing EE value consistent with these orthogonal methods, HILIC appears to be a promising tool for this application. These findings further support the interest of HILIC for rapidly evaluating both mRNA integrity and encapsulation efficiency in just two analytical runs, without the need for extensive sample pretreatment. However, as this study is a preliminary proof-of-concept with qualitative results, further validation and comparison with established methods are needed. Thus, following work using more appropriate stationary phases and a set of well characterized samples is needed to understand the strengths and limitations of these HILIC approaches.

## Conclusion

In this Technical Note, we present for the first time the potential of HILIC as a proof-of-concept tool for the characterization of mRNA in LNP-based drug products. Under harsh conditions, the method destabilizes the LNP structure, releasing the mRNA payload, which is then retained in the stationary phase. The strong retention provided by HILIC allowed mRNA integrity characterization in real drug products. However, the 300 Å pore-size of this commercially available stationary phase technology may be hindering the potential of this new technique. Additional comparisons with orthogonal methods are needed to ensure that HILIC is robust and accurate enough to compete with the established techniques. By developing a second HILIC method using milder conditions, we screened samples for the presence of nonencapsulated mRNA. A cursory assessment of encapsulation efficiency was calculated by measuring the free and total mRNA from two separate injections.

The two methods were developed using Comirnaty and Spikevax vaccines. A qualitative comparison of the integrities obtained with HILIC and with manual disruption was performed, showing similar trends. Encapsulation efficiencies obtained were also comparable to the values reported in the literature. To further evaluate the performance of our methods, we assessed the integrity and encapsulation efficiency of a complex mRNA drug product provided by Sanofi. The results were consistent with those obtained using orthogonal methods, and good resolution for mRNA species of similar sizes was obtained.

In summary, our preliminary findings highlight the potential of this proof-of-concept application of HILIC for direct online disruption of LNPs, mRNA integrity characterization, and evaluation of the encapsulation efficiency. However, no validation has been carried out to determine whether HILIC can compete with established methods. Therefore, this study should be regarded solely as a qualitative proof-of-concept investigation. Additional work is required to validate the results presented here and explore other features such as lipid-adduct formation, stability of LNPs at high flow rates, and the potential benefits of newly developed HILIC stationary phases with larger pore sizes. Finally, limitations inherent to HILIC, such as high adsorption problems, limited robustness, and reduced column lifetime due to the injection of LNPs, will be characterized to evaluate whether HILIC might find its place in quality control environments.

## Supporting Information Available

Description of the integration limits when measuring integrity for disrupted mRNA-LNP samples (PDF)
