# Supplementary material for: Proof of Concept Application of Hydrophilic Interaction Chromatography for Direct Online Disruption of Lipid Nanoparticles, Intact mRNA Analysis, and Measure of Encapsulation Efficiency
Source: Anal Chem. 2025 Apr 1;97(14):7627–32. doi: 10.1021/acs.analchem.5c00565 (PMC12004355; doi:10.1021/acs.analchem.5c00565)
Supplement: Supplementary file 1 — ac5c00565_si_001.pdf [file ac5c00565_si_001.pdf]

SUPPORTING INFORMATION

Proof of Concept Application of Hydrophilic Interaction Chromatography for Direct Online Disruption of Lipid Nanoparticles, Intact mRNA Analysis, and Measure of Encapsulation Efficiency

Jonathan Maurer[1,2,3], Matthew A. Lauber[4], Szabolcs Fekete[5], Mateusz Imiołek[5], Camille Malburet[3], Marc François-Heude[3], Davy Guillarme[1,2,*]

[1] School of Pharmaceutical Sciences, University of Geneva, CMU-Rue Michel Servet 1, 1211 Geneva, Switzerland

[2] Institute of Pharmaceutical Sciences of Western Switzerland, University of Geneva, CMU-Rue Michel Servet 1, 1211 Geneva, Switzerland

[3] mRNA Center of Excellence, Analytical Sciences, Sanofi, 1541 Avenue Marcel Mérieux, 69280 Marcy l'Etoile, France

[4] Waters Corporation, 34 Maple Street, Milford, Massachusetts 01757-3696, United States

[5] Waters Corporation, CMU-Rue Michel Servet 1, 1211 Geneva, Switzerland

Table of content

Figure S1. Integration limits for integrity testing 2

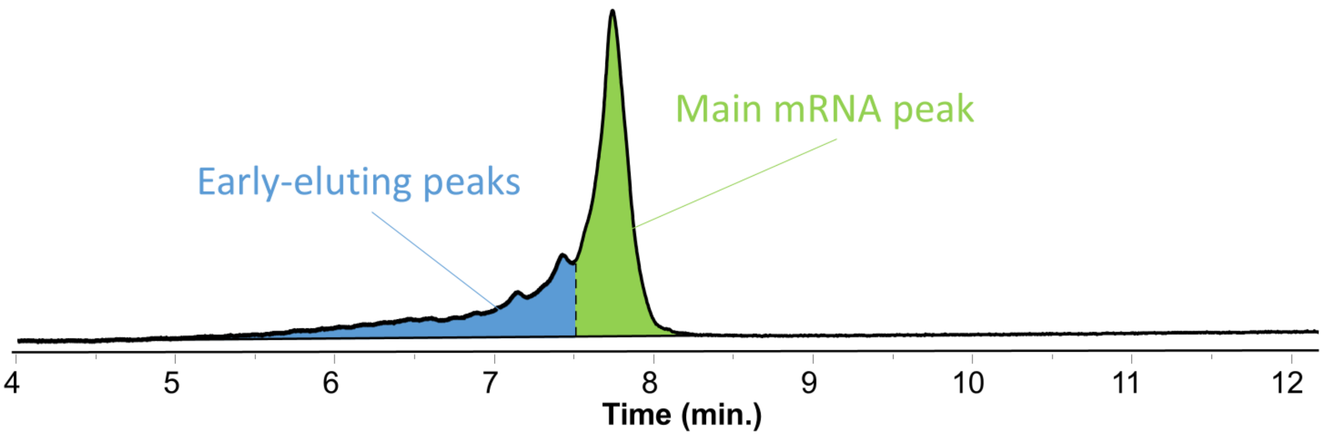

Figure S1. *Integration limits for integrity testing. This chromatogram was obtained for the analysis of the Spikevax vaccine using manual disruption, which consists of a sample diluted 10 times in T4TE20X buffer. Analysis started with 70% ACN for 1 min followed by a gradient from 40% to 30% ACN in 10 min, at 80 °C. Blue peaks represent the early-eluting peaks and green peak represents the main mRNA species. Integrity values are obtained by dividing the green area by the total area (green and blue). To characterize the samples, the same approach was applied to all the samples in the study.*
